# A public health approach to monitoring HIV with resistance to HIV pre-exposure prophylaxis

**DOI:** 10.1371/journal.pone.0272958

**Published:** 2022-08-29

**Authors:** Susan E. Buskin, Richard J. Lechtenberg, Francis A. Slaughter, Joshua T. Herbeck, Roxanne P. Kerani, Matthew R. Golden, Julia C. Dombrowski

**Affiliations:** 1 Public Health – Seattle & King County, Seattle, Washington, United States of America; 2 Department of Epidemiology, University of Washington, Seattle, Washington, United States of America; 3 Department of Global Health, University of Washington, Seattle, Washington, United States of America; 4 Department of Medicine Allergy and Infectious Diseases, University of Washington, Seattle, Washington, United States of America; Chinese University of Hong Kong, HONG KONG

## Abstract

**Background:**

The risk of HIV pre-exposure prophylaxis (PrEP) failure with sufficient medication adherence is extremely low but has occurred due to transmission of a viral strain with mutations conferring resistance to PrEP components tenofovir (TDF) and emtricitabine (FTC). The extent to which such strains are circulating in the population is unknown.

**Methods:**

We used HIV surveillance data to describe primary and overall TDF/FTC resistance and concurrent viremia among people living with HIV (PLWH). HIV genotypes conducted for clinical purposes are reported as part of HIV surveillance. We examined the prevalence of HIV strains with mutations conferring intermediate to high level resistance to TDF/FTC, defining primary resistance (predominantly K65R and M184I/V mutations) among sequences reported within 3 months of HIV diagnosis and total resistance for sequences reported at any time. We examined trends in primary resistance during 2010–2019 and total resistance among all PLWH in 2019. We also monitored resistance with viremia (≥1,000 copies/mL) at the end of 2019 among PLWH.

**Results:**

Between 2010 and 2019, 2,172 King County residents were diagnosed with HIV; 1,557 (72%) had a genotypic resistance test within three months; three (0.2%) had primary TDF/FTC resistance with both K65R and M184I/V mutations. Adding isolated resistance for each drug resulted in 0.3% with primary TDF resistance and 0.8% with primary FTC resistance. Of 7,056 PLWH in 2019, 4,032 (57%) had genotype results, 241 (6%) had TDF/FTC resistance and 15 (0.4% of those with a genotype result) had viremia and TDF/FTC resistance.

**Conclusions:**

Primary resistance and viremia combined with TDF/FTC resistance are uncommon in King County. Monitoring trends in TDF/FTC resistance coupled with interventions to help ensure PLWH achieve and maintain viral suppression may help ensure that PrEP failure remains rare.

## Introduction

Daily pre-exposure prophylaxis (PrEP) with emtricitabine (FTC) and tenofovir (disoproxil fumarate [TDF] or alafenamide [TAF], hereafter included with TDF) reduces the risk of HIV acquisition by >90% with consistent use [1–4]. PrEP is a key component of HIV prevention in the *Ending the HIV Epidemic* initiative [5]. However, case reports provide evidence that, even among individuals who are highly adherent to PrEP, HIV breakthrough infection does occur, albeit rarely, particularly with viral strains resistant to FTC and TDF [6–9]. In a 2021 review article, To and Lee found 10 reported cases of PrEP breakthrough infections, and only three of the 10 were infected with wild-type virus.

The prevalence of HIV strains with resistance to both TDF and FTC has not been well-described among population-based cohorts of newly HIV-diagnosed individuals nor among persons living with HIV infection (PLWH). HIV drug resistance is monitored in King County, Washington (the Seattle metropolitan area) through the National HIV Surveillance System (NHSS) [10, 11]. NHSS includes viral sequences from genotypic tests conducted in routine HIV clinical care. King County is a potential sentinel site for monitoring the emergence of TDF/FTC resistant virus due to high levels of PrEP uptake [12–14]. In 2018, we estimated 47% of men who have sex with men (MSM) at high risk for HIV were taking PrEP [14]. Only New York City and San Francisco, among the 48 counties with the largest HIV burdens in the U.S., had higher rates of PrEP use per total population [15].

The objectives of our study were to (1) estimate the population-level prevalence of and trends in primary TDF/FTC resistance to track the acquisition of circulating resistant strains over time and (2) examine the prevalence of total resistance (primary or acquired) in 2019 including co-occurring resistance and viremia among PLWH to describe people who are potential sources of transmitting resistant strains.

## Methods

### Populations

To examine trends in primary TDF/FTC resistance, we included King County residents newly diagnosed with HIV 2010–2019, excluding individuals who self-reported HIV diagnoses > one year before their diagnoses in NHSS [16]. To examine prevalent TDF/FTC resistance and concurrent viremia, we included PLWH presumed living in King County on 12/31/2019, regardless of the jurisdiction of diagnosis.

### Resistance

We defined HIV strains as “TDF/FTC-resistant” if they contained mutations conferring intermediate to high level resistance to both emtricitabine and tenofovir as interpreted by the Stanford database algorithm [17]. This included K65R, M184I/V, and thymidine analogue mutations (TAMs): M41L, L210W, and T215YF. We also present the frequency of intermediate to high level single drug (isolated TDF and FTC) resistance.

### Primary resistance

TDF/FTC resistance was defined as primary when found ≤ three months from HIV diagnosis. Due to small numbers of PLWH with primary resistance, to examine trends we collapsed the comparison to 2010–2012 versus 2013–2019 due to licensure of the first PrEP medication (comprised of TDF & FTC) in 2012 [18]. We also examined “any level” of primary resistance from possible through higher levels per the Stanford database algorithm.

### Total resistance

The combination of primary and acquired resistance (that identified at any time) is “total TDF/FTC resistance”. A subset of total resistance is *prevalent resistance*, limited to individuals with TDF/FTC resistance identified with their most recent genotypic sequence. Prevalent resistance excludes people who had evidence of drug resistance on ≥ one genotype sequence reported but were without resistance on their most recent genotype (reversion to wild type, with potential archived resistance).

### Viremia

Viremia was defined as a plasma viral load (VL) ≥ 1,000 copies/mL, a more conservative transmission threshold than that reported by Quinn et al (finding no sexual transmissions at <1,500 copies/mL) [19].

### Genotype sequences

Starting in 2003, King County has participated in Centers for Disease Control and Prevention funded projects collecting HIV genotypic sequences [11]. Laboratories report partial *pol* region genotype sequences conducted in routine medical care of PLWH.

### NHSS data

To compare individuals with and without TDF/TFC resistant viral strains, we used NHSS data collected through routine laboratory reporting of HIV diagnostic and care tests (HIV screening tests, CD4+ T cell subsets, and VL results); medical record reviews; partner services interviews; and provider-initiated case reports. Partner services interviews also provided data on PrEP use prior to or at the time of seroconversion. Demographic and clinical characteristics included sex assigned at birth, race/ethnicity, age, HIV risk category, nativity, and VL.

### Statistical analyses

We used *X*^2^ testing (or Fischer’s exact) to examine trends in primary TDF/FTC resistance, compare sociodemographic characteristics by TDF/FTC resistance status, and compare those with and without genotypic sequences reported. These analyses were conducted with SAS (version 9.4, SAS Institute Inc., Cary, NC, USA) and OpenEpi (Version 3.01, Centers for Disease Control and Prevention and Emory University, Atlanta, GA, USA).

### Human subjects

This analysis was determined by the local public health authority (MRG) to be a priority public health surveillance activity and not research requiring IRB review.

## Results

### Primary resistance

Between 2010 and 2019, 2,172 King County residents were diagnosed with HIV. Of these, 1,751 (81%) had ≥ one genotypic sequences reported. Most sequences were obtained within three months (N = 1,557, 89%) or one year (N = 1,679, 96%) of HIV diagnosis. Of sequences obtained within three months of diagnosis, three PLWH (0.19%) had primary TDF/FTC resistance (in 2014, 2016, and 2018). All three individuals had both K65R and M184I/V mutations. An additional 10 PLWH had isolated primary FTC resistance (with HIV diagnoses spanning 2010 through 2019) and 2 had isolated primary TDF resistance (in 2012 and 2016). Primary “any level” TDF/FTC resistance was found in eight PLWH (two in 2010 and 2016, one each in 2012, 2014, 2018, and 2019). Two of the three had used PrEP for > one year, and the third’s PrEP use history was unknown (they declined a partner services interview). Assuming a similar prevalence of TDF/FTC resistance for those recently diagnosed with HIV and without a genotypic sequence reported within three months (N = 607) we estimate one additional primary resistance case, totaling four PLWH with primary TDF/FTC resistance in the decade.

Comparing PLWH diagnosed 2010–2012 versus 2013–2019, there was no trend towards an increase in primary TDF/FTC resistance detected, nor an increase in primary TDF resistance or primary FTC resistance, nor an increase in “any level” TDF/FTC resistance.

### Total TDF/FTC resistance

Of 7,056 PLWH in King County at the end of 2019, 4,040 (57%) had one or more genotypic sequence reported, of whom 241 (6%) had TDF/FTC resistance at any time (primary or acquired). A total of 743 PLWH had FTC resistance (18% of 4,040); 502 (68%) of the 743 had isolated FTC resistance. TDF resistance was found in 286 PLWH (7% of 4,040), 45 (16% of 286) had isolated TDF resistance.

Multiple genotypic sequences were reported for 168 of the 241 (70%) PLWH with TDF/FTC resistance—with up to 11 sequences reported. Of the 168, 82 (49%) did not have prevalent TDF/FTC resistance, or resistance at their most recent genotypic test. And 85 (51%) had at least one sequence reported without resistance to TDF/FTC following one or more sequence with TDF/FTC resistance. Of the 85, the median time to loss of resistance was three years (IQR 1.4–6.9 years).

Of the total 241 PLWH with TDF/FTC resistance, M184I/V mutations were present for 84%, K65R mutations were found in 28% (both including 23% with both M184I/V and K65R). Of the 26 PLWH (11% of 241) with TDF/FTC resistance and with neither major mutation, 77% had > one TAM. The most common TAMs present among the 26 were T215Y/F (18 PLWH, 69% of 26) and M41L (15 PLWH, 58% of 26).

Among PLWH living in King County at the end of 2019 who had one or more sequence reported, the following characteristics were associated with TDF/FTC resistance: male sex at birth (vs. female sex at birth), Non-Hispanic/Latinx White race (vs. all other races or Hispanic/Latinx ethnicity), age 30–39 years at HIV diagnosis (vs. all other age groups), age > 50 years at the end of 2019 (vs. all other ages), MSM (vs. other HIV transmission categories), and U.S.-born PLWH (vs. non-U.S. born) (see Table 1). The median diagnosis year for PLWH with TDF/FTC resistance was 1994, versus 2008 for those without TDF/FTC resistance and 2006 overall. Of the 241 PLWH in 2019 with TDF/FTC resistance at any time, 15 (6%) had a most recent VL in 2019 > 1,000 copies per mL. An additional 20 PLWH with TDF/FTC resistance did not have a VL reported in 2019; however, three-quarters of these (N = 15) had a suppressed VL in 2018 or 2020. The simultaneous occurrence of both viremia and TDF/FTC resistance thus occurred in 15 of 7,056 (0.21%) PLWH. Among 787 people with either FTC or TDF resistance mutations, 62 (8%) had viremia with a VL above 1,000 at the end of 2019.

**Table 1:** People living with HIV in King County, WA at the end of 2019 by genotypic test results indicating tenofovir/emtricitabine (TDF/FTC) resistance.

		Combined TDF/FTC resistance		No higher level resistance to both TDF/FTC (includes single drug or low level resistance)		No genotype		TOTAL	
Total		241	100%	3,791	100%	3,024	100%	7,056	100%
Sex assigned at birth^a^	Male	**223**	**93%**	**3,261**	**86%**	2,645	87%	6,129	87%
	Female	**18**	**7%**	**530**	**14%**	379	13%	927	13%
Race/ ethnicity^b^	White	**138**	**57%**	**1,910**	**50%**	1,682	56%	3,730	53%
	Black	40	17%	784	21%	618	20%	1,442	20%
	Latino/a/x/Hispanic	38	16%	604	16%	404	13%	1,046	15%
	Multi-racial	21	7%	297	8%	145	5%	463	7%
	Asian	**2**	**1%**	**152**	**4%**	151	5%	305	4%
	Pacific Islander	0	··	20	1%	6	<1%	26	<1%
	Native American	2	1%	24	1%	18	1%	44	1%
Age group (age in 2019)	<20	1	<1%	14	<1%	16	1%	31	<1%
	20–29	**2**	**1%**	**280**	**7%**	173	6%	455	6%
	30–39	**7**	**3%**	**818**	**22%**	516	17%	1,341	19%
	40–49	**24**	**10%**	**967**	**26%**	650	21%	1,641	23%
	50–59	**111**	**46%**	**1,111**	**29%**	983	33%	2,205	31%
	60+	**96**	**40%**	**601**	**16%**	686	23%	1,383	20%
Age at HIV infection (years)	<20	4	2%	126	3%	70	2%	200	3%
	20–29	**60**	**25%**	**1,210**	**32%**	914	30%	2,184	31%
	30–39	**131**	**54%**	**1,376**	**36%**	1,150	38%	2,657	38%
	40–49	42	17%	718	19%	622	21%	1,382	20%
	50–59	**2**	**1%**	**285**	**8%**	220	7%	507	7%
	60+	2	1%	76	2%	48	2%	126	2%
HIV risk category^c^	Man who had sex with men (MSM)	**182**	**76%**	**2,390**	**63%**	2,121	70%	4,693	67%
	Person who used injection drugs (PWID)	7	3%	193	5%	90	3%	290	4%
	MSM-PWID	21	9%	417	11%	203	7%	641	9%
	Heterosexual	22	9%	454	12%	303	10%	779	11%
	Other	3	1%	47	1%	42	1%	92	1%
	Unknown	**6**	**2%**	**290**	**8%**	265	9%	561	8%
Nativity	Foreign born	**40**	**17%**	**896**	**24%**	733	24%	1,669	24%
	U.S.-born, including unknown nativity	**201**	**83%**	**2,895**	**76%**	2,291	76%	5,387	76%
Viremia	Viral load ≥ 1,000	15	6%	246	6%	40	1%	301	4%
	VL < 1,000	203	84%	3,164	83%	2624	87%	5,991	85%
	No viral load reported in 2019	23	10%	381	10%	360	12%	764	11%
HIV diagnosis date	Before 2012	**233**	**97%**	**2,629**	**69%**	2,203	73%	5,065	72%
	2012–2015	**4**	**2%**	**614**	**16%**	415	14%	1,033	15%
	2016–2019	**4**	**2%**	**552**	**15%**	402	13%	958	14%
Resistance mutations	M184I/V	219	91%	504	13%	n/a	n/a	723	10%
	K65R	86	36%	1	<1%	n/a	n/a	87	1%

^a^ Of the 7,056 prevalent cases, 62 were transgender women (1% of 6,129 people assigned male at birth) and 5 were transgender men (1% of 927 people assigned female gender at birth). None of the transgender men and four of the transgender women had TDF/FTC resistance (8% of 50 with sequences reported).

^b^ Individuals of Hispanic/Latinx ethnicity are excluded from other categories.

^c^ MSM include transgender women who have sex with men; heterosexuals include presumed heterosexuals, defined as women who had sex with men and denied a history of injection drug use.

**Bold typeface** indicates statistically different prevalence of TDF/FTC resistance in this row versus all others at p <0.05.

## Discussion

We found that 0.2% of individuals newly diagnosed with HIV in King County had primary TDF/FTC drug resistance and, similarly, 0.2%, of PLWH living in King County in 2019 had both TDF/FTC resistance and viremia. We found no evidence of increasing primary TDF/FTC resistance during the 10 year period 2010–2019 despite widespread adoption of PrEP among local MSM, with nearly half of MSM at high-risk for HIV using PrEP by 2018 [12, 14].

To our knowledge, this is the first population-based report describing trends in and prevalence of circulating HIV strains that could potentially overcome PrEP to result in transmission. Two earlier studies reported resistance to either TDF or FTC separately but not jointly. In a four-site study of virally unsuppressed MSM with HIV infection in the U.S., researchers reported 16% with resistance to TDF or FTC [20]. Younger et al also looked at both primary and acquired resistance to TDF and FTC in a collaborative including eight Canadian cohorts and presented the results for each but not TDF/FTC together [21]. They found primary resistance to TDF in 1.5% and resistance to FTC in 0.4% and 5-year rates of acquired resistance at 0.7 and 3.3% respectively [20].

A key limitation of our analysis is that the likelihood of transmission of a virus with TDF/FTC resistance from a viremic individual to a sexual partner taking PrEP is unknown. To estimate the proportion of PLWH who might be at risk for transmitting “PrEP resistant” HIV strains, we focused on those with a VL threshold of ≥ 1,000 copies/mL. This definition may have been either too stringent (e.g. missing people with viremia at times other than the result included in this analysis) or not stringent enough (e.g. capturing people with a transient high VL and including archived resistance, unlikely to be transmitted) [22]. We also did not have any information about the sexual behavior of people with resistance mutations and viremia, who may not actually have been at risk of transmitting HIV. Without complete histories of antiretroviral use, we may have misidentified acquired resistance as primary resistance, although at least one report indicated this is not common [23]. The timing of a resistance test may also have contributed to misclassification of primary resistance (if the test was too long after seroconversion) or misclassification of prevalent resistance (if the resistance test was some time ago). Nonetheless, these limitations likely had no significant impact on our conclusion that co-occurring TDF/FTC resistance and viremia is rare in King County, and likely elsewhere. Although our analysis was limited to one geographic area, it is an area with a high prevalence of PrEP use, which would be expected to enrich the chances of finding resistance. However, it is also an area with high levels of viral suppression, which undoubtedly negates the risk of transmission regardless of resistance mutations in archived virus.

A question that arises from this work is what interventions can or should be implemented to prevent PrEP resistance-associated HIV transmission. We demonstrated that public health surveillance data can be used to identify individuals potentially at increased risk for transmitting HIV to partners on PrEP, and, given few people in this category, that outreach to these individuals is feasible. The primary goal of such outreach should be to support achieving viral suppression, consistent with the goals of other public health efforts. As part of such efforts, the person contacting the individual to assist with re-engagement in care and treatment could counsel the individual about their genotype and caution that partners on PrEP may not be protected from HIV acquisition. This would support individual autonomy in decisions about HIV treatment and prevention.

## Conclusions

Our findings are reassuring that (1) primary TDF/FTC resistance is rare and has not increased in the setting of widespread PrEP use among over 2,000 newly diagnosed individuals over 10 years and (2) that individuals with high levels of viremia and with TDF/FTC resistance were uncommon in this population-based cohort of over 7,000 PLWH. Health departments collecting sequence data can replicate this approach to monitor TDF/FTC resistance in their jurisdictions.

## Supporting information

S1 File (DOC)
