# Supplementary material for: A public health approach to monitoring HIV with resistance to HIV pre-exposure prophylaxis
Source: PLoS One. 2022 Aug 29;17(8):e0272958. doi: 10.1371/journal.pone.0272958 (PMC9423671; doi:10.1371/journal.pone.0272958)
Supplement: S1 File — (DOC) [file pone.0272958.s001.doc]

Memorandum

To: Susan Buskin, PhD, MPH, Co-Principal Investigator

From: Matthew Golden, MD, MPH; Director, PHSKC HIV/STD Program

Date: August 29, 2026

Re: Surveillance determination for “A public health approach to monitoring HIV with resistance to HIV pre-exposure prophylaxis”

I am writing to support and authorize an analysis of surveillance data collected and maintained by the HIV/STD Program at Public Health – Seattle & King County. The analysis, “A public health approach to monitoring HIV with resistance to HIV pre-exposure prophylaxis” led by you (Dr. Susan Buskin) as a University of Washington faculty member and epidemiologist for Public Health – Seattle & King County. Your team is authorized to use data from the King County portion of the National HIV Surveillance System (NHSS) for 2010-2019.I am authorizing you as the Principal Investigator for this surveillance project to conduct this work under my authority as a public health official and HIV Control Officer for King County.

NHSS is a CDC-funded surveillance system, and as such it is considered to be non-research. Per the revised Common Rule for the conduct of human subjects research, a secondary analysis of NHSS data, if it meets [three criteria described in 45 CFR 46.102](https://www.hhs.gov/ohrp/regulations-and-policy/requests-for-comments/draft-guidance-activities-deemed-not-be-research-public-health-surveillance/index.html) does not merit IRB review. This analysis meets all three criteria:

- “The activity must be a public health surveillance activity (defined as collecting, testing, analyzing, and using information or biospecimens to improve public health and prevent disease; providing timely and useful evidence, and enabling public health authorities to be more effective in their efforts to protect and promote public health);”

**NHSS is clearly public health surveillance.**

- “The activity must be conducted, supported, requested, ordered, required, or authorized by a public health authority; and”

**In your role as an epidemiologist for the HIV/STD Program at Public Health – Seattle & King County, you have the authority to conduct this analysis. As the HIV Disease Control Officer and Director of the HIV/STD Program, this memo serves as my concurrence.**

- “The activity must be limited to that necessary to allow a public health authority to identify, monitor, assess, or investigate potential public health signals, onsets of disease outbreaks, or conditions of public health importance (including trends, signals, risk factors, patterns in diseases, or increases in injuries from using consumer products).”

**The results from this analysis will provide our health department and community with a contemporary snapshot of the prevalence and characterization of resistance to the components of pre-exposure prophylaxis (PrEP), which is our most potent tool for HIV prevention. These data are of critical importance for informing public health efforts and response.**

Please contact me with any questions or concerns. I can be reached via email at golden@uw.edu or phone at 206-744-6829. Thank you.

CC: Julia Dombrowski, MD, MPH Co-Principal Investigator
